# Real-Time Monocular Skeleton-Based Hand Gesture Recognition Using 3D-Jointsformer

**DOI:** 10.3390/s23167066

**Published:** 2023-08-10

**Authors:** Enmin Zhong, Carlos R. del-Blanco, Daniel Berjón, Fernando Jaureguizar, Narciso García

**Affiliations:** Grupo de Tratamiento de Imágenes (GTI), Information Processing and Telecommunications Center, ETSI Telecomunicación, Universidad Politécnica de Madrid, 28040 Madrid, Spain; carlosrob.delblanco@upm.es (C.R.d.-B.); daniel.berjon@upm.es (D.B.); fernando.jaureguizar@upm.es (F.J.); narciso.garcia@upm.es (N.G.)

**Keywords:** human–computer interaction (HCI), hand gesture recognition (HGR), real-time processing, skeleton-based hand gesture recognition, 3D-CNNs, transformers, self-attention mechanism

## Abstract

Automatic hand gesture recognition in video sequences has widespread applications, ranging from home automation to sign language interpretation and clinical operations. The primary challenge lies in achieving real-time recognition while managing temporal dependencies that can impact performance. Existing methods employ 3D convolutional or Transformer-based architectures with hand skeleton estimation, but both have limitations. To address these challenges, a hybrid approach that combines 3D Convolutional Neural Networks (3D-CNNs) and Transformers is proposed. The method involves using a 3D-CNN to compute high-level semantic skeleton embeddings, capturing local spatial and temporal characteristics of hand gestures. A Transformer network with a self-attention mechanism is then employed to efficiently capture long-range temporal dependencies in the skeleton sequence. Evaluation of the Briareo and Multimodal Hand Gesture datasets resulted in accuracy scores of 95.49% and 97.25%, respectively. Notably, this approach achieves real-time performance using a standard CPU, distinguishing it from methods that require specialized GPUs. The hybrid approach’s real-time efficiency and high accuracy demonstrate its superiority over existing state-of-the-art methods. In summary, the hybrid 3D-CNN and Transformer approach effectively addresses real-time recognition challenges and efficient handling of temporal dependencies, outperforming existing methods in both accuracy and speed.

## 1. Introduction

In recent years, there has been a significant increase in research on novel devices and techniques for human–computer interactions (HCIs). In particular, hand gesture interfaces have gained popularity in various applications, including smart homes, health monitoring, virtual reality, automobile equipment control, and sign language translation [1,2,3,4]. Human communication heavily relies on gestures as a natural and intuitive means of expression. Therefore, developing accurate and efficient hand gesture recognition systems can significantly enhance how humans interact with computers, devices, and virtual environments. Such advancements can lead to more immersive experiences in virtual reality (VR) and augmented reality (AR) applications [5,6], and revolutionize gaming experiences by enabling gesture-based controls [7]. Moreover, it can improve human–robot interactions in areas such as healthcare, manufacturing, and assistance robotics. Additionally, hand gesture recognition has the potential to enhance accessibility for individuals with disabilities by offering alternative and inclusive input methods [8,9].

Existing technologies for hand gesture recognition can be classified into two main categories: sensor-based and vision-based methods. Sensor-based methods rely on wearable devices to capture motion data and recognize hand gestures. These devices include sensors such as accelerometers [10], inertial measurement units (IMUs) [11], optical sensors [12], and surface electromyography (sEMG) devices [13]. This approach has demonstrated high accuracy in recognizing dynamic hand gestures. However, it requires significant preprocessing to obtain clean data and employs hand-crafted feature techniques, which are time-consuming. Additionally, it imposes significant constraints on real-life situations as these devices need to be constantly attached to the body or hands.

Camera-based methods offer a more natural way to interact with human–machine interfaces, eliminating the need for on-body devices. Existing studies have utilized various sources of data for hand gesture recognition tasks, such as RGB, depth, infrared, and optical flow, used either independently or in combination with each other [14,15]. Processing continuous video streams of these data are computationally intensive due to their high intradimensional and multidimensional nature. Alternatively, specific camera-based devices can acquire skeletal data, which is more computationally efficient since it considers only the position of the hand joint coordinates, resulting in a significantly lower data volume compared to the number of image pixels. These methods also alleviate limitations regarding varying illumination changes and common problems of self-occlusion in hand gestures. Furthermore, the increasing accessibility of cost-effective human action capture systems, such as Microsoft Kinect [16], Intel RealSense camera [17], or Leap Motion controller [18], has facilitated the acquisition of skeletal data. However, these are proprietary systems that cannot be modified, improved, or adapted to specific situations. Under such circumstances, the hand recognition system must include a pose/skeleton recognition module specifically designed to convert a video stream into a skeleton stream. Additionally, it is desirable for such conversion to be achieved using a monocular camera to simplify the hardware requirements of the system.

Regardless of the skeleton recognition method used, analyzing the temporal dependencies of a sequence of skeletons offers more advantages than analyzing a sequence of images. However, skeleton sequences can exhibit significant intra-class and inter-class variability, making it challenging to learn discriminative features for accurate hand gesture classification. Additionally, understanding the temporal dynamics of skeleton sequences is crucial for capturing motion patterns and transitions between different hand poses. Modeling long-term dependencies and capturing subtle temporal changes in hand keypoints can also pose challenges.

Motivated by this, some authors have applied CNN-based methods to hand skeleton sequences to learn hand posture variations and hand movements in different branches and subsequently fuse the learned features. However, Convolutional Neural Networks (CNNs) may not efficiently address temporal dependencies compared to Transformers. To better model long-range temporal changes, other authors have introduced Transformer-based methods. Most of the employed embedding strategies are not efficient as they directly model the pairwise relations of the entire input skeleton sequence, encompassing both spatial and temporal dimensions. This approach overlooks the importance of spatial interactions among distinctive local joints and the significance of capturing short-term temporal dynamics, which are essential for recognizing hand motion patterns. In summary, the main research gap lies in the fact that existing methods fail to achieve real-time hand gesture recognition without compromising accuracy. This issue can be attributed to the lack of more efficient embedding strategies capable of capturing spatial interactions among joints in short-term temporal dynamics, as well as the absence of more effective methods to analyze long-term temporal dependencies in skeleton sequences.

The main objective of the proposed work is to achieve real-time hand gesture recognition and address the identified research gaps by introducing an efficient hybrid neural network. This network comprises a Local Spatio-Temporal Embedding module implemented using 3D-CNN and a Long-Term Embedding module implemented using a Transformer. The Local Spatio-Temporal Embedding module is designed to analyze both the spatial relationships between subgroups of hand joints and the temporal evolution of those relationships over time. This local feature extraction proves beneficial for recognizing fine-grained hand gestures. Implementing the Long-Term Embedding module using the Transformer architecture, the network aims to better capture long-range dependencies, thereby refining the learned representations by attending to the global temporal dynamics and interactions between subgroups of hand joints. The experimental results demonstrate that the proposed hybrid neural network achieves high recognition accuracies of 95.49% and 97.25% after evaluation on Briareo and Multimodal Hand Gesture datasets, respectively. This work contributes to the advancement of skeleton-based hand gesture recognition by effectively addressing temporal dependencies and efficiently capturing spatial interactions, leading to improved performance and potential applications in various fields, such as VR, AR, and human–robot interactions.

The novelty of this research lies in its innovative hybrid neural network architecture that combines 3D-CNN and Transformer models. By fusing these two techniques, the system can leverage both spatial and temporal information contained in the embeddings, addressing the limitations of traditional methods. Previous sensor-based approaches often suffered from high computational requirements, time-consuming preprocessing, and restricted practicality due to the need for on-body devices. On the other hand, vision-based methods face challenges in efficiently modeling temporal dependencies. However, the proposed hybrid neural network overcomes these limitations.

The main contributions can be summarized as follows:

- **Hybrid Neural Network Design:** The proposed approach presents a novel hybrid neural network for skeleton-based hand gesture recognition, efficiently combining a 3D-CNN to infer high-level semantic skeleton embeddings with a Transformer-based model that utilizes a self-attention mechanism to capture long-term dependencies of the previous sequence of skeleton embeddings. It should be noted that the skeleton embeddings contain partial information in the spatial and time domain; that is, an embedding includes information from a subset of skeleton nodes along a limited time span.
- **Real-time Efficiency:** A major breakthrough achieved in this research is the real-time capability of the system. Thanks to the carefully designed neural network architecture, the entire system can efficiently run on a Computer Processing Unit (CPU), eliminating the need for specialized and resource-intensive hardware. This achievement is a crucial step toward practical applications of hand gesture recognition in real-world scenarios.
- **Competitive Performance:** The proposed method demonstrates competitive performance on the main hand gesture recognition benchmarks. By effectively capturing local spatial relationships and long-term temporal dependencies, the hybrid neural network outperforms the state-of-the-art accuracy by 4.19% on the Briareo dataset.

## 2. Related Work

Existing skeleton-based action recognition works [19,20,21,22] have inspired the closely related area of skeleton-based hand gesture recognition [23,24,25,26]. Thus, raw skeletal data are usually converted into pseudo-images, graph data, or time series to be efficiently processed. Early works proposed hand-crafted descriptors for hand skeleton joints [23,24]. The usual information managed by these descriptors was the distance among hand joints, the geometric shape of the hand, and the direction, translation, and rotation of the joints over time. However, they offered a limited representation of the underlying skeletons, ignoring complex, long, and not evident relationships among the hand joints. Deep learning approaches solve this problem by estimating adaptive representations and descriptors for the target application, which can be divided into different families: Convolutional Neural Networks (CNNs), Recurrent Neural Networks (RNNs), 3D Convolutional Neural Networks (3D-CNNs), Graph Convolutional Networks (GCNs), and Transformer-based methods.

**Methods based on 1D Convolutional Neural Networks (CNNs)**. These methods take sequences of skeleton joint coordinates as input and perform 1D convolution along the temporal dimension. For instance, Devineau et al. [24] proposed a parallel Convolutional Neural Network to process each hand joint independently as a separate channel. They then used a late fusion strategy to obtain a final hand feature vector for classification. However, this approach presents some inconveniences. If some joints are missing or incomplete, parallel CNNs may struggle to effectively handle such data. Moreover, the computational complexity is notably higher than that of sequential or fused architectures, as independent CNNs are required for each joint.

**Long Short-Term Memory (LSTM)**. This is a type of Recurrent Neural Network (RNN) that is well suited for modeling temporal sequences of joint coordinates. However, LSTMs do not inherently handle spatial information or inter-joint coordinates. This limitation can be addressed by integrating CNNs before LSTMs or RNNs. Previous works often converted skeleton coordinates into pseudo-images and skeleton sequences into video streams to apply convolutional layers to skeleton sequences. However, such input conversions may not fully exploit the locality of convolutional layers [20], as skeletal data are fundamentally non-Euclidean spatial data. Therefore, mapping joint coordinates into pseudo-images could lead to a loss of spatial correlation among them. In [27,28], the authors proposed a system based on a combination of CNN and LSTM. The CNN focuses on detecting spatial patterns related to the location of skeletal joints in a 3D space. Subsequently, the LSTM is employed to capture spatio-temporal patterns associated with the evolution of the 3D coordinates of skeleton joints over time.

Narayan et al. [29] proposed a system based on a concept very similar to those in [27,28]. They utilized a multi-channel CNN followed by an LSTM to extract spatio-temporal features from 3D hand joints for hand gesture recognition. Although LSTMs are proficient in capturing short-term dependencies, they might struggle to learn and model highly complex long-term dependencies in sequences. This limitation becomes significant in cases where the skeleton data contain intricate patterns or gestures that depend on actions occurring relatively far in the past.

**Methods based on 3D Convolutional Neural Networks (3D-CNNs)**. These methods are particularly interesting for analyzing dynamic hand gestures, as they can capture both local spatial and temporal characteristics. Liu et al. [30] proposed a 3D-CNN-based method to extract skeleton dynamics. Specifically, the authors encoded hand postures and hand movements into separate streams and then applied a 3D-CNN and a 2D-CNN to each stream individually. This approach may have limitations in fully capturing the complex interactions between hand posture and hand movement. Additionally, the method’s performance may be affected by the handcrafted separation of information into different streams.

Mohammed et al. [31] proposed a novel approach for skeleton-based hand gesture recognition using a multi-model ensemble. Their method involves the combination of multilayer 3D-CNN, temporal Convolutional Neural Networks, and convolutional LSTM networks. These components process the input features, and the resulting representations are then concatenated in a late-fusion manner to produce the final feature vector for classification. Combining multiple models in a multi-model ensemble network introduces additional complexity and computational overhead. Training and deploying such a combined system could require more resources and time compared to using a single model. In their paper, the authors reported an improved accuracy of only 1% higher for the multi-model ensemble network than that for the single model using 3D-CNN. The trade-offs between the accuracy gains and computational complexity should be considered.

To the best of the authors’ knowledge, there are fewer studies exploring the full potential of 3D-CNNs for analyzing skeleton data compared to their more common utilization for RGB-image-based hand gesture recognition. For example, Chen et al. [32] designed a multiscale 3D-CNN to be applied to multimodal hand gesture data to make the final classification. They obtained an accuracy on the Briareo dataset of 91.3% on RGB data and 92.7% on depth data. Dhingra et al. [33] introduced attention blocks and residual networks between non-consecutive 3D convolution layers to build deeper and more discriminative networks. In [34], 3D convolutional layers were used in a ResNet backbone to classify between gesture and no gesture classes. Both works [33,34] leveraged 3D convolutional layers within a ResNet backbone for gesture classification; while this approach can effectively capture spatio-temporal features from the input data, it may suffer from the limited modeling of long-range dependencies. As hand gestures often involve complex temporal dynamics, it is crucial to consider methods that can efficiently capture and exploit long-term dependencies between gesture frames.

**Graph Convolutional Networks (GCNs)**. These have gained significant attention in recent years for recognizing skeleton-based hand gestures [35,36,37,38,39]. These methods treat the skeleton as a graph, with joints as nodes and their spatial connections as edges. Graph Convolutional Networks (GCNs) are capable of learning spatial dependencies among joints and propagating information through the skeletal graph. However, they heavily rely on the defined graph topology, making it crucial to construct an accurate and optimal graph representation.

In a study by Li et al. [26], they defined a fixed hand graph structure as the input for the GCN. This design limits the model’s capacity to learn dependencies between unlinked joints since the graph convolution layers only operate among the connected hand joints. To address this limitation, subsequent works have introduced improvements in the design of the topology of the hand graph. For example, in [36,40], the authors designed a dynamic hand graph that adjusts dynamically based on different hand gestures. However, this approach may have limited generalization ability for unseen hand gestures as it heavily relies on the learned hand graph structure. Similar to CNN-based methods, GCNs also face challenges in modeling and capturing long-range temporal information.

**Transformer-based methods**. Transformer architectures excel at capturing long-term dependencies, in contrast to previous methods that mainly focused on short-term information. Therefore, they are particularly well suited for analyzing long skeleton sequences that represent hand gestures. Surprisingly, while there is a substantial body of research on Transformer-based architectures in skeleton-based action recognition [41,42,43,44], their application for skeleton-based hand gesture recognition remains comparatively limited.

The main drawback of a pure Transformer architecture is the simplistic modeling of spatial data, specifically the joint relationships inside the skeleton. This is typically accomplished by using a fully connected layer to create an embedding that globally encodes all the joints in the skeleton [45]. As a result, information exchange and dependencies among neighboring joints are either not considered or not adequately addressed. In the pure Transformer-based network proposed by Liu et al. [46], the aim is to capture local and global spatio-temporal features of the skeleton sequence. However, they manually group the joints of each finger into the same subgroup and then apply a linear projection layer to each subgroup to obtain the final embedding. This local embedding strategy converts the skeleton sequence into a predefined structure, leading to the same limitation as using the local linear projection mentioned above. For color image data, D’Eusanio et al. [14] propose using ResNet-18 as the backbone to extract spatial characteristics from each input frame and the Transformer encoder to perform temporal analysis between frames. The model obtains a classification accuracy of 90.6% for the 12 gesture classes in the Briareo dataset. ResNet-18 is a relatively shallow architecture compared to deeper variants like ResNet-50 or larger models like ResNet-101. The limited capacity of ResNet-18 could affect its ability to capture complex spatial features in color image data, potentially resulting in suboptimal performance.

Despite the advances made in previous studies, effectively capturing both spatial and temporal information in hand skeleton sequences for gesture recognition remains a challenging task. To address these specific issues, this work proposes a novel method, named 3D-Jointsformer, that combines the power of 3D-CNN and Transformers. The proposed method is specifically designed for skeleton-based gesture recognition, where input sequences consist of hand-skeleton data. By leveraging the strengths of both architectures, the proposed hybrid approach aims to effectively encode spatial and temporal information, achieving real-time hand gesture recognition without compromising accuracy.

## 3. Proposed Method

**Figure 1:**
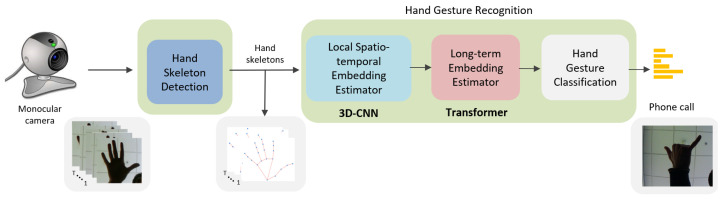
Block diagram of the proposed 3D-Jointsformer. The proposed system aims to be applied in real-world scenarios where the input consists of a sequence of RGB frames captured by a monocular camera. These sequences of images are subsequently processed by the Hand Skeleton Detection component. The obtained hand skeleton keypoints are then fed into the hand gesture recognition component to determine the final hand gesture class. The hand gesture recognition component is composed of a Local Spatio-Temporal Embedding Estimator, which computes Local Spatio-Temporal Embeddings from subsets of keypoints, and a Long-Term Embedding Estimator, which models long-range temporal relations of the output embeddings. These components are implemented using 3D-CNN and Transformer, respectively.

The proposed system, called 3D-Jointsformer, enables real-time recognition of hand gestures from color video sequences using a standard CPU, in contrast to other approaches that require specialized hardware like Graphical Processing Units (GPUs). The system consists of two modules (see Figure 1): Hand Skeleton Detection and hand gesture recognition. In the first module, Hand Skeleton Detection, a set of 3D key points or joints representing the hand skeleton is inferred from every image. This results in a sequence of hand skeletons obtained from the video stream. The second module, hand gesture recognition, processes and classifies this sequence into one of the predetermined hand gestures. This recognition module consists of three blocks. The first block, the Local Spatio-Temporal Embedding Estimator, employs a specially designed 3D-CNN architecture to compute local embeddings that encode subsets of skeleton joints within a short time span. These embeddings serve as local high-level semantic representations of the hand skeleton sequence. The second block, the Long-Term Embedding Estimator, utilizes a Transformer-based architecture to efficiently capture long-term dependencies among all the previous local embeddings. The output is a final and holistic feature-based representation of the hand skeleton sequence. The third and final stage, hand gesture classification, globally combines all the information from the previous holistic feature to infer the class of the involved hand gesture. Overall, 3D-Jointsformer offers real-time hand gesture recognition capabilities without compromising accuracy, using readily available CPU resources.

### 3.1. Hand Skeleton Detection

The Hand Skeleton Detection module is based on [47] and integrates a hand detector and skeleton estimator. The hand detector estimates the location of hand instances in real time across a wide range of scales and appearances with high accuracy using just color imagery. Then, the hand skeleton estimator infers the hand keypoints from previous hand image regions. Given that *T* is the length of the sequence of hand keypoints and *V* is the total number of hand joints, a gesture sample, $n_{th}$, is encoded by a sequence of vectors, $X_{n}=\left\{H_{t}^{n}|t=1,2,\ldots ,T\right\}$, where every vector $H_{t}^{n}=\left\{x_{v,t}^{n},y_{v,t}^{n},z_{v,t}^{n}∥v=1,\ldots ,V\right\}$, which represents a set of 3D joint coordinates encoding a hand skeleton. In addition, to be able to process gestures of variable temporal length, either because they belong to different categories of gestures or are performed by different individuals, an interpolation in the time domain is performed over the sequences of hand skeletons of different lengths to consider a fixed-length hand sequence of 32 skeletons.

### 3.2. Hand Gesture Recognition

The hand gesture recognition module estimates the category of the performed gesture from a sequence of 3D skeletons through three different stages. First, the Local Spatio-Temporal Embedding Estimator computes local embeddings that encode subsets of skeleton joints within short time spans, resulting in high-level semantic representations of subparts of a hand skeleton sequence. These embeddings are obtained using a 3D-CNN neural network, which is a modified implementation of SlowFast Networks, initially conceived for Video Recognition [48]. The original SlowFast architecture comprises two streams: the Slow Pathway and the Fast Pathway. The Slow Pathway processes frames at a lower frame rate and aims to capture broader motion patterns and context changes in the video. On the other hand, the Fast Pathway operates at a higher frame rate and is designed to capture fast and subtle motion patterns. The backbone architecture of the original SlowFast combines 3D convolutional layers for the Slow Pathway and 2D convolutional layers for the Fast Pathway. Finally, the outputs from both the Slow and Fast Pathways are fused together to obtain the final prediction. While this architecture has shown great success in recognizing actions performed by the entire body, it may not be ideal for capturing the fine-grained movements specific to hand gestures. Therefore, several key modifications have been designed and implemented to adapt and enhance the existing framework for Local Spatio-Temporal Embeddings of hand skeleton sequences.

**Figure 2:**
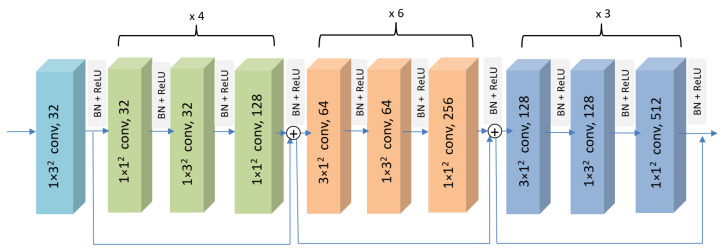
Overview of the proposed Local Spatio-Temporal Embedding Estimator. By utilizing 3D convolutions, the model can handle the time-based evolution of skeleton joints, capturing their temporal dynamics over successive frames. The dimensions of the kernels in the convolutional blocks are represented as $\left(T\times S^{2},C\right)$, where *T* and *S* denote the temporal and spatial strides, respectively, and *C* represents the number of channels. The choice of $\left(T\times S^{2}\right)$ for the kernel dimensions is influenced by the SlowFast and other 3D-CNN-based architectures commonly used for video recognition. Experimental results verify that processing the input skeleton sequences with these temporal and spatial resolutions facilitates the learning of hierarchical representations of the gestures.

Regarding the nature of the hand skeleton, four modifications were introduced to the original architecture. The first modification is to adopt only the Slow Pathway stream from the original architecture to fulfill the purpose of this local embedding module, which is to compute the Local Spatio-Temporal Embedding from subsets of joints. By using only the Slow Pathway, the overall computational complexity of the model is reduced, facilitating real-time operation. The second modification reduces the depth of the network by using fewer convolutional blocks, considering that the number of joints is much less than the number of pixels in an image. This helps avoid overfitting problems associated with excessive network parameters while reducing computational complexity. The third modification involves reducing the width of the network by using fewer channels for similar purposes. The fourth modification is to use a smaller kernel size, which reduces the receptive field, aiming to focus on capturing more local spatial information. Figure 2 depicts the specific sizes and channels. The overall architecture of the modified system aims to capture finer spatial features of the input skeleton sequence. It does so by processing frames at a lower temporal resolution in the earlier layers of the network while increasing the temporal resolution in the deeper layers to capture fine-grained temporal dynamics and hand motion information. The detailed architecture of the proposed 3D-CNN network is shown in Figure 2.

Based on the optimal trade-off between computational efficiency and performance, the final setting of the proposed lightweight Local Spatio-Temporal Embedding Estimator is composed of four CNN blocks. The first block, also known as the stem layer, processes an input tensor of C×T×V, representing a hand skeleton sequence, where *C* stands for channel, *T* stands for temporal, and *V* stands for spatial dimensions. The kernel size applied is $1\times 3^{2}$, considering neighborhoods of three joints since hand gestures are finer than whole-body actions and operate on smaller spatial dimensions. Note that the temporal stride is set to one to preserve the temporal resolution, while the spatial stride is three, allowing the model to capture global patterns of the hand joints inside each frame. The second block stacks several 3D-CNN layers with kernel sizes of $1\times 3^{2}$ and $1\times 1^{2}$, focusing on processing local spatial joint information without mixing the temporal aspect. The last two blocks, 3 and 4, introduce a kernel size of $3\times 1^{2}$, combining temporal information and increasing the temporal receptive field to capture a wider temporal context.

For all blocks, batch normalization (BN) and rectified linear unit (ReLU) layers are applied after each convolutional layer, helping to regularize the model. Moreover, residual connections are adopted after each block to alleviate the problem of vanishing gradients and stabilize the network training. Consequently, the output of the 3D-CNN backbone is a feature map of size $S\in R^{TxD}$, where *D* represents the embedding dimension of the model.

**Figure 3:**
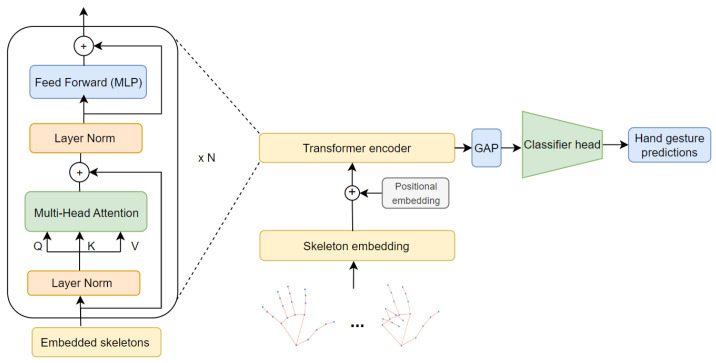
The Long-Term Embedding Estimator implemented by the Transformer neural network. Each encoder block in the Transformer consists of a Multi-Headed Attention (MHA) layer and a Feed-Forward Neural Network (FFN). MHA enables the model to attend to different parts of the input sequence simultaneously, allowing it to learn complex relationships and dependencies between different joints in the hand skeleton. The FFN further refines the representations by applying non-linear transformations. To enhance the training process and stabilize the learning, layer normalization is applied before each layer in the encoder block. A global pooling layer (GAP) is applied to aggregate the representations across all time steps. Finally, the aggregated feature representation is fed to the classifier head, which predicts the final gesture.

The second stage, known as the Long-Term Embedding Estimator, focuses on inferring long-term temporal interactions from the previous feature map, *S*, which contains local spatio-temporal joint skeleton information. To achieve this, a Transformer neural network [49] is employed, consisting of multiple Transformer encoder blocks that determine the depth of the model. These encoder blocks refine and improve the representations by capturing increasingly complex patterns and long-range dependencies. Each encoder block utilizes a self-attention mechanism to draw long-term dependencies among the spatio-temporal joint skeleton embeddings. Specifically, the Long-Term Embedding Estimator comprises an initial embedding layer followed by *N* Transformer encoder blocks (as illustrated in Figure 3). The best performance was achieved by employing *N* = 2 blocks of the Transformer encoder. This result confirms our hypothesis that the spatio-temporal embeddings obtained from the previous Local Spatio-Temporal Embedding Estimator represent high-level representations of the input skeleton sequence. Adding more Transformer encoder blocks would propagate unnecessary information through the network, leading to an insignificant improvement in accuracy.

The embedding layer takes every component of the feature map, *S*, as a token and adds a positional embedding proposed by Vaswani et al. [49], which adopts the sine and cosine functions with different frequencies.

Defining *PE* as the positional embedding; *p* and *i* as the position of the embedded skeleton component and the total number of components of *S*, respectively; $d_{model}$ as the dimension of the output embedding space; and sin and cos as the sine and cosine functions, respectively, the positional embedding equation is represented as follows:

(1) $$\begin{array}{c} PE\left(p,2_{i}\right)=\sin(\frac{p}{10,000^{\frac{2_{i}}{d_{model}}}})\\ PE\left(p,2_{i}+1\right)=\cos(\frac{p}{10,000^{\frac{2_{i}}{d_{model}}}}) \end{array}$$

Next, the resulting embeddings are passed through the attention blocks, where a normalization layer is applied, and three distinct new embeddings, namely, Query (*Q*), Key (*K*), and Value (*V*), are computed using fully connected layers. Subsequently, the dot product, $QK^{T}$, is calculated to determine the pairwise similarities between different components of *S*, the input sequence. To ensure proper scaling, the dot product is normalized by a factor of $\sqrt{d_{k}}$, where $d_{k}$ represents the dimension of each embedding (*Q* or *K*). The resulting values are referred to as attention scores, and they contain information about the affinity of each spatio-temporal joint skeleton embedding with respect to the others in the sequence. These attention scores are further processed using the softmax function, which enables them to represent the importance of each embedding in relation to the others. Finally, the attention scores are used as weights to integrate the most relevant spatio-temporal contextual information into each local joint skeleton embedding. This process is illustrated in Equation (2), where Att represents the attention function.

(2) $$\begin{array}{c} Att\left(Q,K,V\right)=\mathrm{softmax}(\frac{QK^{T}}{\sqrt{d_{k}}})\cdot V \end{array}$$

(3) $$\begin{array}{c} MHA\left(s\right)=\mathrm{Concat}\left(Att_{1}\left(s\right),\ldots ,Att_{8}\left(s\right)\right)\cdot W^{o} \end{array}$$

The attention block illustrated in Equation (2) is applied in a parallel fashion, known as Multi-Head Attention (MHA) [49], by dividing the input embedding vector into parts, which are processed by different heads or attention blocks. Each head learns a different representation from different perspectives, which are then linearly combined into one final feature vector. Specifically, eight heads are used, each with a dimension of 64. The attention scores obtained from each head ($Att_{1},\ldots ,Att_{8}$), where *s* denotes the input sequence of the corresponding Transformer encoder block, are then concatenated using the *Concat* operation. This concatenated output is projected into a vector of dimension $d_{model}=512$ using the matrix of learned parameters represented by $W^{o}$, as shown in Equation (3). The number of attention heads, the embedding size of the model, and the head dimension were determined based on the settings commonly applied in the literature and tuned by the experimental observations performed in this work, taking into account the trade-off between model size and performance.

Each Transformer encoder block is composed of Multi-Head Attention (MHA) and Feed-Forward Neural Network (FFN) blocks (Equations (4) and (5)). Given the input sequence, $s_{n-1}$, the feature representation, $s_{n}^{\prime }$, obtained by MHA is then passed to the FFN block, which globally aggregates all the spatio-temporal skeleton information. As shown in Equation (5), the FFN comprises a Multilayer Perceptron (MLP) and Layer Normalization (LN). The MLP is composed of two fully connected layers, followed by a Gaussian Error Linear Unit (GELU) activation function. The output of the Transformer encoder block is added to the subsequent one, and the dimension of the embedding remains consistent across all the encoder blocks.

(4) $$\begin{array}{c} s_{n}^{\prime }=MHA\left(LN\left(s_{n-1}\right)\right)+s_{n-1}\;\;\;n=1\ldots N \end{array}$$

(5) $$\begin{array}{c} s_{n}=MLP\left(LN\left(s_{n}^{\prime }\right)\right)+s_{n}^{\prime }\;\;\;n=1\ldots N \end{array}$$

In the final stage, the classifier head comprises a fully connected layer followed by a softmax layer, which calculates a probability distribution across all hand gesture classes. The predicted gesture is then determined to be the one with the highest probability.

## 4. Results and Discussion

This section provides a comprehensive explanation of the experimental setup used to assess the performance of the proposed real-time hand gesture recognition system. It includes the evaluation metrics and provides details about the hardware and software configurations to ensure reproducibility and facilitate further research in the field.

### 4.1. Experimental Settings, Metrics, and Datasets

The proposed system was implemented using the PyTorch deep learning framework. It was trained and evaluated on a server equipped with an Intel Core i7-4790 with a 3.30 GHz CPU and 32 GB of memory, along with an Nvidia Titan Xp (12 GB) GPU. The proposed model was trained with a batch size of 32 video samples for a total of 100 epochs. The Adam optimizer was chosen to minimize the cross-entropy loss due to its adaptive learning rate property. The initial learning rate was set to 1 $\times \;10^{-3}$, with a weight decay of 1 $\times \;10^{-4}$. Furthermore, gradient clipping was applied to stabilize the training process by imposing an $L_{2}$ norm constraint on the gradient values. Specifically, if ||g||≥c, then ||g|| is rescaled to *c*, where ||g|| represents the $L_{2}$ norm of the gradient, *g*, and c is the threshold for the maximum norm, empirically set to 40. To avoid overfitting, we also applied the “drop attention” strategy introduced by Zehui et al. [50] to regularize attention weights in Transformer networks. This dropout rate of attention was set to 0.2, which randomly drops 20% of the columns of the attention weight matrix. This value was found to best generalize the model after conducting several experiments.

Regarding metrics, prediction accuracy was used to evaluate the proposed model using the standard top-1 accuracy. Equation (6) shows the general mathematical expression for the top-k accuracy, where $y_{i}$ is the true label of the *i*th sample and ${\hat{y}}_{i,j}$ represents the corresponding predicted value for the *i*th sample and the *j*th highest probability score.

(6) $$\begin{array}{c} Acc_{top-k}\left(y,\hat{y}\right)=\frac{1}{n_{samples}}\sum_{i=0}^{n_{samples}-1}\sum_{j=1}^{k}1\left({\hat{y}}_{i,j}=y_{i}\right) \end{array}$$

**Figure 4:**
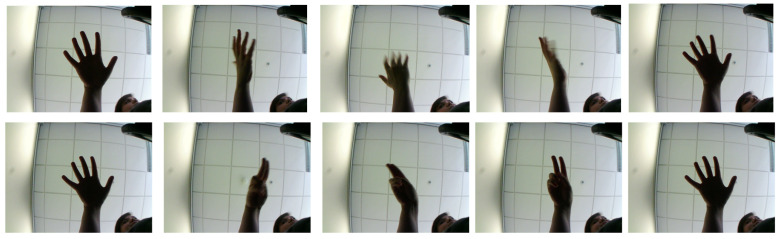
Examples of gestures in the RGB images from the Briareo dataset.

The Briareo [51] and Multimodal Hand Gesture [52] datasets were used to evaluate the proposed hand recognition system. They contain RGB and IR images, unlike other related datasets, such as the DHG 14/28 [23] and SHREC’17 track [53], easing the integration of the system into a wide range of applications as no specialized sensors are required. The Briareo dataset is focused on dynamic hand gesture recognition and contains 12 different classes of gestures performed with the right hand by 40 different people (33 men and 7 women). Each action is repeated three times and captures the entire motion of the gesture. The entire dataset contains 1440 sequences divided into training, validation, and test sets, with 65%, 15%, and 20% for each set, respectively. The authors of the Briareo dataset proposed this specific data distribution, which has been widely adopted by other works to facilitate fair and meaningful comparisons between different methods. The gestures were designed to facilitate interaction between the driver and the vehicle information system. Figure 4 shows an example of the RGB images containing different dynamic hand gestures in the Briareo dataset. The conditions of acquisition are quite challenging since the illumination is very low, the hand was visually blurred in several frames, and the shape of the fingers is not well appreciated.

### 4.2. Results on Briareo Dataset

The proposed system, 3D-Jointsformer, is compared with other state-of-the-art methods on the Briareo dataset using RGB data in Table 1. C3D is a 3D Convolutional Neural Network originally proposed by Tran et al. [54] for video classification. It is composed of a backbone with five 3D convolution layers, each of which is followed by a pooling layer. Then, the classification head is composed of two fully connected layers and a softmax layer.

The CNN-based architecture proposed by D’Eusanio [55] is based on a modified DenseNet-161 architecture to adapt a 2D-CNN to a continuous input stream. The architecture is composed of several dense transition blocks, with 161 layers in total. A 2D-CNN architecture does not consider the temporal context and, therefore, cannot effectively capture the hand motion dynamics over multiple frames.

ResNet-18 + Transformer [14] is conceptually most similar to the proposed method since it uses a 2D-CNN backbone based on ResNet-18 to extract spatial features at the frame level and a Transformer to model the long-range temporal information of the spatial features extracted by the backbone. However, the 2D-CNN only exploits the spatial information of the input sequence, unlike the proposed 3D-CNN backbone that computes a local spatio-temporal skeleton feature map, where each component encodes information about a subset of joints along a short interval of time. Thus, the posterior Transformer architecture can effectively capture long-term dependencies among different subsets of joints across all time intervals.

Multiscale 3D-CNN [32] proposes another 3D-CNN-based backbone, Inflated 3D ConvNet, to extract spatio-temporal features from input sequences in a multimodal data fusion framework that combines RGB and depth data. Observe that the proposed method achieved the best accuracy score, 95.49%, outperforming the other methods. The second best, Multiscale 3D-CNN, achieves an accuracy of 91.3%, almost 4.2% less than 3D-Jointsformer. Notice also that the algorithm ResNet-18 + Transformer, the one reported as conceptually most similar to 3D-Jointsformer, is not only significantly less accurate than 3D-Jointsformer but also than Multiscale 3D-CNN. This proves that the neural network architecture of 3D-Jointsformer is definitely superior due to the proposed 3D-CNN architecture since it effectively encodes subsets of joints (not just independent joints, nor all the skeleton) in short-time intervals (not just one-time steps, nor all the hand gesture period), which are then processed by the Transformer that captures long-range dependencies via the attention mechanism, integrating global information from the refined spatio-temporal features extracted by the 3D-CNN. Slama et al. [56] proposed a hybrid network in which they combine a spatial Graph Convolutional Network and a Transformer graph encoder for skeleton-based hand gesture recognition. By employing this configuration oriented to operate in real time, they have an accuracy of 83.34% compared to the previous Graph Convolutional Network, which achieved an accuracy of 78.31%. Despite this advancement, the performance of their approach still falls significantly short of the accuracy achieved by the proposed 3D-Jointsformer at 95.49%.

**Figure 5:**
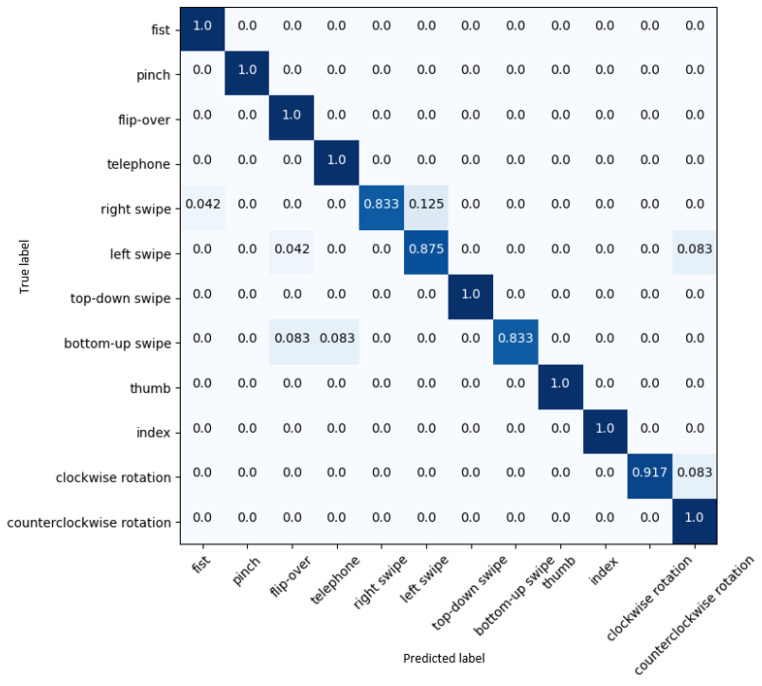
Confusion matrix results on the Briareo dataset.

Additionally, a fivefold cross-validation was conducted on the Briareo dataset to mitigate the effects of data randomness and ensure the consistency of the proposed 3D-Jointsformer’s performance. The average top-1 accuracy over the five folds of the dataset is 95.21 ± 0.89%, which verifies the model’s performance and robustness. The confusion matrix of 3D-Jointsformer shown in Figure 5 allows an analysis of the system’s accuracy per hand gesture. The values on the diagonal represent the normalized percentage of gestures correctly classified by the model, indicating a robust and consistent accuracy score. The proposed 3D-Jointsformer has demonstrated accurate recognition in 8 out of the 12 hand gesture classes present in the Briareo dataset. However, certain hand gesture classes, such as clockwise rotation, right swipe, bottom–up swipe, and left swipe, inherently pose more complexity and challenges for precise detection. This difficulty is due to the temporal symmetry and near-identical spatial localization of the hand keypoints in those closely related gestures. Despite these challenges, the 3D-Jointsformer achieved respectable accuracy rates, correctly identifying 91.9% of the clockwise rotation gesture, 87.5% of the left-swipe gesture, and 83.3% of the right-swipe and bottom–up-swipe gestures.

### 4.3. Ablation Study on Briareo Dataset

An ablation study is presented to measure the influence of the embedding strategy for the input skeleton sequences on the final system performance. The proposed 3D-CNN strategy is compared to a standard and widely used linear projection composed of two fully connected layers followed by a GELU non-linear layer, which extracts a D-dimensional skeleton feature vector per video frame used as tokens for the Transformer. Table 2 shows the accuracy results of both embedding strategies. Observe that the proposed 3D-CNN-based embedding used in the 3D-Jointsformer significantly improves the accuracy by 7.53%. The main reason is that the linear embedding does not consider the local spatial–temporal correlations among the joints.

Another ablation study was carried out to measure the impact of using a Transformer to capture long-term relationships by comparing the strategy adopted in a 3D-Jointsformer with a 3D-CNN network followed by Global Temporal Average Pooling plus a fully connected layer. Table 3 shows the results, revealing that the Transformer block can further improve the accuracy by 1.74%.

Regarding computational cost, 3D-Jointsformer is very lightweight and efficient, with only 8.8 M parameters and 0.17 GFLOPs, which achieves a top-1 accuracy of 95.49%, as previously shown. Thus, it can operate in real time for hand gesture recognition applications. Moreover, it can operate not only in GPU real time but also in CPU. More specifically, the 3D-Jointsformer can process 2790 skeletons/s and 2569 skeletons/s for GPU and CPU, respectively. To ensure a consistent comparison, the metrics reported and the evaluation format are calculated, as described in the referenced paper [51]. It is important to note that the inference time mentioned does not account for the additional processing time required for Hand Skeleton Detection. Additionally, Table 4 shows the inference time results per frame of 3D-Jointsformer and C3D, revealing that 3D-Jointsformer is much faster than C3D.

### 4.4. Ablation Study on the Multimodal Hand Gesture Dataset

The recognition capabilities of the proposed method were also evaluated on the Multimodal Hand Gesture Dataset (MMHGD) [52]. The dataset consists of infrared imagery comprising 16 static hand gesture classes acquired by a Leap Motion device. In total, 25 subjects (8 women and 17 men) participated in recording the dataset. Each gesture has 20 instances (repetitions) per subject, performed in different locations in the image. The dataset was split into train, validation, and test set, with 60%, 20%, and 20% for each set, respectively.

The results, as reported in Table 5, compare the two different system configurations presented in Table 2. Observe that, similarly to the previous dataset results, the Transformer network captures better long-term dependencies, reaching a better accuracy (0.5% higher). Nonetheless, the margin of improvement is less than with the previous dataset. This is due to the fact that the temporal information in static hand gestures (the ones in the MMHGD dataset) is less relevant than in dynamic ones (those in the Briareo dataset).

**Figure 6:**
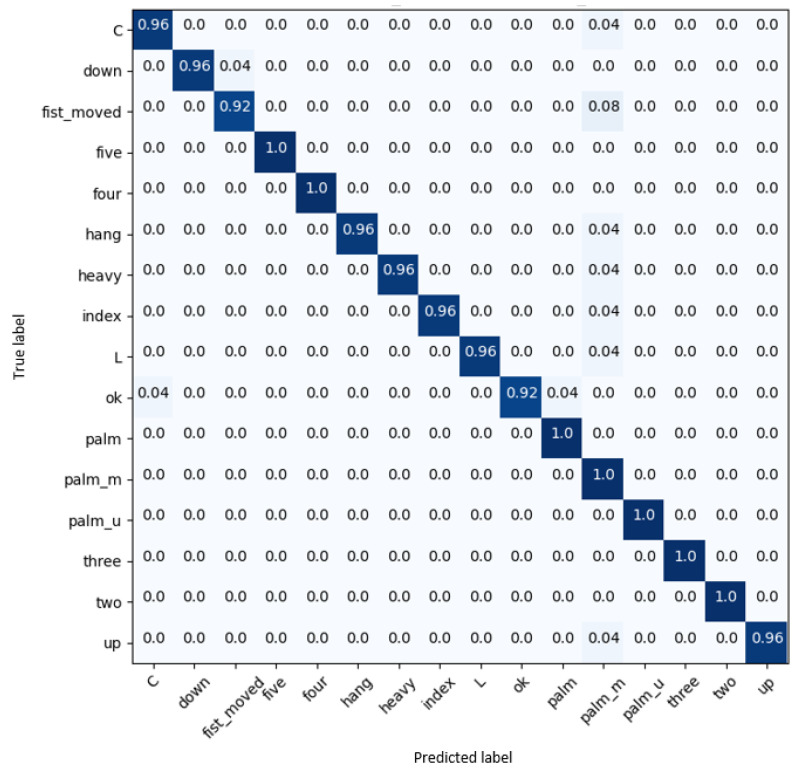
Confusion matrix of results on the Multimodal Hand Gesture Dataset (MMHGD).

Figure 6 presents the confusion matrix of 3D-Jointsformer, where it can be observed that it correctly classifies gestures with very high accuracy in all classes.

## 5. Conclusions

In conclusion, this work introduces a novel hybrid neural network for skeleton-based hand gesture recognition, effectively capturing both local spatio-temporal information and long-term dependencies. The primary objective was to develop a real-time model using only the CPU without sacrificing accuracy. The proposed architecture combines a Local Spatio-Temporal Embedding Estimator utilizing 3D-CNN to capture local patterns and a Long-Term Embedding Estimator based on a Transformer to model sequential dependencies. One of the model’s notable achievements lies in its real-time operation, which addresses the long-standing challenges of rapid data processing in many real-world scenarios. Through careful network design and computational efficiency optimization, the model achieves real-time performance without compromising accuracy. The experimental results on two publicly available datasets demonstrate the superiority of the proposed hybrid neural network over state-of-the-art methods, achieving accuracy rates of 95.49% on the Briareo dataset and 97.25% on the Multimodal Hand Gesture dataset. For future studies, real-world deployment will be addressed by conducting experiments and evaluations of the model’s performance in real-world settings. Feedback should be gathered to assess the model’s usability and user experience, ensuring it meets the needs of the intended applications. This work lays a strong foundation for advancing skeleton-based hand gesture recognition systems with broad implications in human–computer interactions, gaming, robotics, and assistive technologies. 

## Figures and Tables

**Table 1:** Comparison of the recognition accuracy of the proposed system against other state-of-the-art methods on the Briareo Dataset.

Method	Year	Top-1 Acc (%)
C3D [51]	2019	72.2%
CNN-based [55]	2020	83.3%
ResNet-18 + Transformer [14]	2020	90.06%
Multiscale 3D-CNN [32]	2022	91.3%
STr-GCN [56]	2023	83.34%
3D-Jointsformer (**Ours**)	**2023**	**95.49**%

**Table 2:** Effect of different embedding strategies for the input skeleton sequences on the Transformer architecture.

Model Configuration	Top-1 Acc (%)
Transformer with linear embedding	87.96%
Transformer with 3D Convolutional embedding	95.49%

**Table 3:** Comparison of the accuracy in two settings: 3D-CNN followed by a Global Temporal Average Pooling (GTAP) and 3D-CNN followed by a Transformer encoder.

Model Configuration	Top-1 Acc (%)
3D-CNN + Global Temporal Average Pooling	93.75%
3D-CNN + Transformer encoder	95.49%

**Table 4:** Comparison of the inference time results.

Model	Hardware Architecture	Inference Time (ms)
Briareo (C3D)	CPU (Intel i7-6850K, 64 GB)	4010 ± 240
	GPU (Nvidia 1080 Ti)	1.96 ± 0.49
	GPU (Nvidia Titan X)	1.87 ± 0.77
Ours (3D-Jointsformer)	CPU (Intel Core i7-4790, 32 GB)	0.486 ± 0.016
	GPU (Nvidia Titan Xp)	0.448 ± 0.007

**Table 5:** Comparison of the performance of different model configurations on the MMHGD dataset.

Model Configuration	Top-1 Acc (%)
3D-CNN + Global Temporal Average Pooling	96.75%
3D-CNN + Transformer Encoder	97.25%

## Data Availability

Not applicable.

## Abbreviations

The following abbreviations are used in this manuscript:

AR	Augmented Reality
BN	Batch Normalization
CNN	Convolutional Neural Network
CPU	Computer Processing Unit
3D	Three Dimensional
3D-CNN	3D Convolutional Neural Network
GCNs	Graph Neural Networks
GPU	Graphics Processing Unit
HGR	Hand Gesture Recognition
HCI	Human–Computer Interaction
FC	Fully Connected
FFN	Feed-Forward Neural Network
GELU	Gaussian Error Linear Unit
LN	Layer Normalization
LSTM	Long Short-Term Memory
MHA	Multi-Head Attention
MLP	Multilayer Perceptron
PE	Positional Embedding
ReLU	Rectified Linear Unit
RNN	Recurrent Neural Network
VR	Virtual Reality
